# A psychoeducational intervention reduces the need for anesthesia during radiotherapy for young childhood cancer patients

**DOI:** 10.1186/1748-717X-3-17

**Published:** 2008-06-04

**Authors:** Sonja Haeberli, Michael A Grotzer, Felix K Niggli, Markus A Landolt, Claudia Linsenmeier, Roland A Ammann, Nicole Bodmer

**Affiliations:** 1 University Children's Hospital of Zurich, Department of Oncology, Steinwiesstr. 75, 8032 Zurich, Switzerland; 2 University Children's Hospital of Zurich, Department of Psychosomatics and Psychiatry, Steinwiesstrasse 75, Zurich, Switzerland; 3 University Hospital Zurich, Department of Radio-Oncology, Raemistr. 100, 8032 Zurich, Switzerland; 4 University Children's Hospital of Berne, Division of Pediatric Hematology-Oncology, Inselspital, 3010 Berne, Switzerland

## Abstract

**Background:**

Radiotherapy (RT) has become an important treatment modality in pediatric oncology, but its delivery to young children with cancer is challenging and general anesthesia is often needed.

**Methods:**

To evaluate whether a psychoeducational intervention might reduce the need for anesthesia, 223 consecutive pediatric cancer patients receiving 4141 RT fractions during 244 RT courses between February 1989 and January 2006 were studied. Whereas in 154 RT courses corresponding with 2580 RT fractions patients received no psychoeducational intervention (group A), 90 RT courses respectively 1561 RT fractions were accomplished by using psychoeducational intervention (group B). This tailored psychoeducational intervention in group B included a play program and interactive support by a trained nurse according to age to get familiar with staff, equipment and procedure of radiotherapy.

**Results:**

Group A did not differ significantly from group B in age at RT, gender, diagnosis, localization of RT and positioning during RT. Whereas 33 (21.4%) patients in group A got anesthesia, only 8 (8.9%) patients in group B needed anesthesia. The median age of cooperating patients without anesthesia decreased from 3.2 to 2.7 years. In both uni- and multivariate analyses the psychoeducational intervention significantly and independently reduced the need for anesthesia.

**Conclusion:**

We conclude that a specifically tailored psychoeducational intervention is able to reduce the need for anesthesia in children undergoing RT for cancer. This results in lower costs and increased cooperation during RT.

## Background

In pediatric oncology, radiotherapy (RT) alone or in combination with surgery, has become an important treatment option for achieving local tumor control. However, administering RT to children requires a great deal of cooperation [1]. To facilitate RT, many hospitals routinely use sedation or general anesthesia for all young children [2,3]. This demands infrastructure and equipment and may also cause disturbances of daily routine, higher costs, and side-effects in the child [4,5]. Interactive intervention concepts for performing invasive and non-invasive procedures without general anesthesia in children have already been designed for some procedures like magnetic resonance imaging, electroencephalography, or bone marrow aspiration [6-12] and are also increasingly applied in pediatric oncology. They include cognitive distraction, behavioral rehearsal, or multimodal intervention packages [13-16]. Some reports have also shown that interactive psychoeducational interventions can help children to cooperate for RT [1,4,5,17,18]. To our knowledge, however, no study included a direct comparison of supported and unsupported patients in the same institution. Therefore, the purpose of this study was to evaluate the efficacy of a targeted psychoeducational intervention program in decreasing the need for anesthesia in a large group of unselected pediatric oncology patients receiving RT.

## Methods

A series of 223 pediatric cancer patients treated at the University Children's Hospital of Zurich who received RT between February 1989 and January 2006 was included in a retrospective study. These 223 patients underwent 244 RT courses with a total of 4141 RT fractions. Curative RT according to the treatment regimen (median 24 Gray, range 4.5–68 Gray), as well as palliative RT (median 21 Gray, range 4.5–50 Gray) for alleviation of pain were incorporated. Until February 1999, 154 RT courses corresponding to 2580 RT fractions were applied with routine care but without psychoeducational intervention. Since then, patients in 90 RT courses corresponding to 1561 RT fractions received an individually tailored psychoeducational intervention. The intervention consisted of talks with the patient and his family about practical aspects of the upcoming RT procedure and an age-based careful explanation and practice of the RT procedure. Aim of this multimodal support was an age-appropriate preparation for the procedure. Implementally, picture books explaining the procedure, playful inclusion of toys, and a reward systems using beads as tokens for every accomplished RT session were used [17,19,20]. Attendance at least for the preparing computer tomography, RT simulation and the first RT session as well as weekly visitations during the RT procedures was accomplished. This support before and during RT was always given by one of two specially trained nurses and required about 5 – 7.5 hours per patient. On average, they met the patient five times. The procedure of planning and administering RT didn't differ from that of group A. If the patient didn't cooperate and the RT session was not feasible, a second attempt to accomplish RT without anesthesia was arranged. For still uncooperative patients anesthesia was used. This included general anesthesia or sedation by intravenous, rectal, or oral medication. Chloral hydrate (50–80 mg/kg, rectal administration) was the first choice for sedation.

The association of the psychoeducational intervention and of six predefined clinical variables with the need for anesthesia was analyzed using uni- and multivariate Poisson regression, with the number of RT fractions as rate multiplier. These clinical variables were time point and age at start of RT, sex, diagnostic group (CNS tumor, extracranial solid tumor, leukemia), localization of RT (head versus other localizations), and positioning during irradiation (supine versus other). For multivariate analysis, stepwise forward variable selection was chosen. LogXact 6 software was used (Cytel Software Corp., Cambridge, MA, USA).

## Results

Group A and B did not differ significantly in respect of sex, age, total dose and times of irradiation, diagnosis, site of irradiation and positioning, thus indicating that the two groups of patients are comparable from a clinical point of view. In total, anesthesia was needed in 41 (16.8%) of 244 RT courses. This proportion was significantly lower in group B (8 of 90 = 8.9%) than in group A (33 of 154 = 21.4%; *P *= 0.015, table 1). This difference remained significant when corrected for several variables potentially associated with the need for anesthesia, including the year of RT as an indicator of changes in the environment (multivariate *P *= 0.018; table 2). A sensitivity analysis regarding only the first RT course per patient fully confirmed these findings (multivariate odds ratio [OR], 0.74; 95% confidence interval [CI], 0.57–0.97; *P *= 0.027).

**Table 1:** Clinical Characteristics of Group A (no intervention) and Group B (intervention)

	**Group A** **No Intervention**		**Group B** **Intervention**		***P***
		
	**N**	**(%)**	**N**	**(%)**	
**Number of RT courses**	154	63.1%	90	36.9%	
**Number of RT fractions**	2580	62.3%	1561	37.7%	
**Sex **					
Male	103	66.9%	61	67.8%	
Female	51	33.1%	29	32.2%	
**Age**					
Median	7.9		8.1		
Mean	9		8.9		
Range	0.0 – 19.0		1.6 – 19.1		
**Anesthesia**					
Yes	33	21.4%	8	8.9%	p = 0.015
No	121	78.6%	82	91.1%	
**Age of those with anesthesia (Years)**					
Median	3.2		2.7		p < 0.001
Mean	3.2		2.7		
Range	0.0 – 12.0		1.6 – 4.3		
Standard Deviation	2.1		0.9		
**Total dose of irradiation (Gray)**					
Median	24.0		20.0		
Mean	28.9		31.2		
Range *	4.0 – 59.4		6.0 – 68.0		
Standard Deviation	16.8		19.0		
**Total times of irradiation**					
Median	12.5		12.0		
Mean	16.8		17.3		
Range	1.0 – 54.0		3.0 – 36.0		
Standard Deviation	10.3		10.6		
**Diagnostic group**					
CNS tumor	31	20.1%	25	27.8%	
Leukemia/Lymphoma	67	43.5%	43	47.8%	
Solid tumor	56	36.4%	22	24.4%	
**Site of irradiation**					
Cranial irradiation	98	63.6%	56	62.2%	
Extracranial irradiation	56	36.4%	34	37.8%	
**Positioning**					
Prone position	19	12.3%	19	21.1%	
Supine position	129	83.8%	64	71.1%	
Other position	6	3.9%	7	7.8%	
**Intent of Radiotherapy**					
Curative	132	85.7%	83	92.2%	
Palliative	22	14.3%	7	7.8%	

Calculation of clinical characteristics is based on number of RT courses and not RT fractions.

In 6 patients in group A and 4 patients in group B low total irradiation dosages of 4 to 6 Gray were applied for pain reduction for palliative treatment, and 4.5 and 6 Gray in a curative setting in two infants with neuroblastoma in Group B.

**Table 2:** Results of Uni- and Multivariate Logistic Regression of Clinical Variables on Need of Anesthesia

	**Univariate Poisson regression**		**Multivariate Poisson regression**	
**Potential Predictor**	**Odds Ratio (95% CI)**	***P***	**Odds Ratio (95% CI)**	***P***

Age (odds ratio per year)	0.52 (0.49 to 0.54)	< 0.001	0.58 (0.55 to 0.62)	< 0.001
Gender	0.53 (0.26 to 1.10)	0.090	0.81 (0.67 to 0.99)	0.039
Diagnostic group (reference: CNS tumor)				
Leukemia	33.7 (16.6 to 68.4)	< 0.001	18.6 (9.1 to 38.2)	< 0.001
Solid tumor	36.8 (18.2 to 85.9)	< 0.001	17.0 (8.1 to 35.7)	< 0.001
Site of irradiation (head vs. others)	0.83 (0.68 to 1.00)	0.049	1.35 (1.04 to 1.77)	0.027
Positioning (head vs. others)	6.97 (4.58 to 10.60)	< 0.001	-	NS
Psychoeducational support	0.37 (0.29 to 0.47)	< 0.001	0.74 (0.57 to 0.95)	0.018
Time of irradiation (odds ratio per year)	0.91 (0.89 to 0.93)	< 0.001	-	NS

NS indicates not significantly associated with need of anesthesia in multivariate analysis.

**Figure 1:**
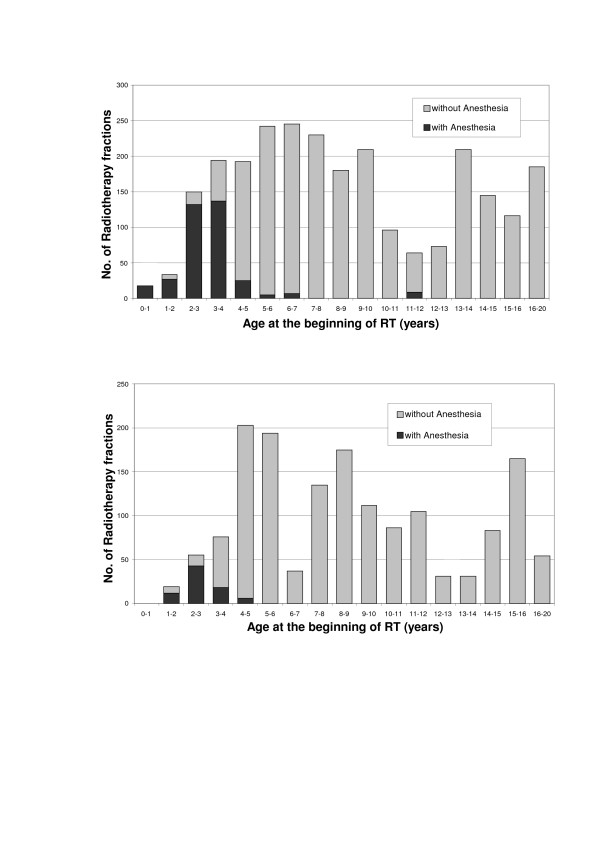
**Age distribution and need for anesthesia**. Age at start of radiotherapy and need for anesthesia in 164 radiotherapy courses without (A) and 90 courses with psychoeducational intervention (B). Black bar indicates RT with anesthesia, grey bar indicates RT without anesthesia.

The psychoeducational intervention reduced the median age of patients needing anesthesia from 3.2 (range 0.0 – 12.0) to 2.7 (range 1.6 – 4.3) years (Figure 1; OR for children below 5 years of age, 0.37; 95% CI, 0.29–0.47; *P *< 0.001). Overall, more girls needed anesthesia than boys (18/80 girls vs. 23/164 boys). Interestingly, the support seemed to be more effective in girls than in boys, however; while without support 33.3% of all female patients needed anesthesia, the anesthesia rate could be reduced with support to only 3.4%. In contrast, there was only a slight decrease of anesthesia in boys due to application of support: While 15.5% of all male patients without support needed anesthesia, the rate was still 11.5% with support.

The costs for our intervention during a RT course, including in median six hours caring by the qualified nurse and material costs, were on average 220 USD for each patient. The median costs for anesthesia during a RT course were estimated to be 4600 USD for an average of 11 sedations or narcosis per accomplished RT. Psychoeducational support was offered to all patients of group B regardless their age and RT planning details. In considering the above mentioned costs for anesthesia the costs for group A were 151800 USD for 33 anesthesia procedures, corresponding to 985 USD per patient (n = 154). In group B the costs for 8 procedures with anesthesia were 36800 USD and the costs for psychoeducational support for all 90 patients were 19800 USD, which means 630 USD per patient in group B. Therefore, the psychoeducational intervention reduced the costs by 36%.

## Discussion

In the current study, an individually tailored psychoeducational intervention was effective in reducing the need for anesthesia in pediatric oncology patients receiving RT. To our knowledge this study is one of the largest investigations of pediatric cancer patients receiving RT.

To exclude an effect of the changes of the medical environment and procedures during the 17 years of the study the influence of the treatment year was included in our multivariate analysis and was shown not to be significant. Therefore, the changes in RT instruments, better facilities or personnel changes in the time between February 1989 and January 2006 didn't influence the decreasing need of anesthesia over the time. Our anesthesia rate of 8.9% (of all RT courses), respectively 5.1% (of all RT fractions) in the intervention group is lower than in comparable patient groups in the literature, which show rates from 10.8% to 39.0% [1,4,5,18]. Interestingly, nearly two thirds of patients younger than 5 years in group B (14 of 22 versus 15 of 45 in group A, Figure 1) were able to cooperate without anesthesia in the often frightening environment, whereas many centers routinely use general anesthesia in this age group [2,3]. One would expect RT to the head to be more frightening to the child than a more distant radiation field [2,17]. It is interesting that the psychoeducational intervention helped our patients to cooperate without anesthesia even in this difficult RT area. Surprisingly, our intervention was clearly more effective in girls than in boys. This might be attributable to the female nurse specialists applying the psychoeducational intervention. On that account, it would be important in the future to find more gender-specific strategies to cope with cancer. Another reason for this gender-related difference might be that girls are initially more afraid of RT than boys. It has previously been shown, that girls are more emotionally suffering from the diagnosis of cancer than boys [21]. Another explanation could be that girls in general are more motivated for such interventions than boys. Other factors such as cultural influences or the social situation of the family were not analyzed here.

In estimating the benefit of a psychoeducational support for RT, it is important not only to consider the age but also the site of irradiation, the planned positioning, and the gender of the patient. The need of intensive support is greater for example in a young girl expecting RT to the head or RT in a prone position than for an older boy with RT to a distant field or a supine positioning.

Clearly, the good outcome in our intervention group might also have been influenced by a more active participation of the parents, who gained a better understanding of the planned procedure and therefore were better able to assist their child during RT; this might be the most important benefit of our intervention in toddlers younger than 2 years. Furthermore, increased awareness by the medical team could also have influenced the decreased need for anesthesia in the supported group. Our psychoeducational intervention was a multimodal support package. This procedure not only facilitates a better understanding of the procedure, but also enables the child to share his situation with his parents and his play dolls [13].

Reducing the use of anesthesia offers several advantages to the child, their families, and the hospitals. One of them is the reduction in costs, as shown by several studies [1,4,5]. We were also able to show a reduction of costs by 36% due to our intervention. As the costs for the support per patient are so much lower compared to anesthesia, the only slight overall reduction of costs is astonishing. Psychoeducational support was offered to all patients undergoing RT regardless their age and RT planning details, however. Therefore many patients, especially those in the age group older than 10 years, received support although they might have been able to cooperate without anesthesia without being supported. In older patients, intervention is more helpful in reducing stress and anxiety levels and not the need of anesthesia, but this was not evaluated in our study. In order to further reduce costs the intervention could be offered only those patients who benefit most, which means for example girls or patients with RT in a prone position or RT to the head. But beside the cost reduction the immeasurable better understanding and comfort also of those patients with the assumed unnecessary support has to be kept in mind. Other studies were also able to show reduction of medical risks, of child distress, improved quality of care, and patient/family satisfaction by reducing the anesthesia rate [16]. However, these parameters were not included in our study, nor were treatment side effects as such, including fatigue and post-treatment nausea. The question as to whether a decreased use of anesthesia might improve the children's quality of life, or whether anesthesia might be superior to the traumatizing experience of RT, remains open.

## Conclusion

In summary, our findings confirm the benefits of a psychoeducational intervention in preparing young pediatric cancer patients receiving radiotherapy. It has been shown in a large series of 244 consecutive oncology patients that psychoeducational intervention was able to significantly reduce the need of anesthesia during RT. Even young patients were able to cooperate for radiation without anesthesia, which resulted in a reduction of costs and an increased cooperation during RT.

## Competing interests

The authors declare that they have no competing interests.

## Authors' contributions

NB made substantial contributions to conception and design of the study and was mainly involved in drafting the manuscript. MAG had idea of this targeted psychoeducational intervention during radiotherapy and was also supervisor of this study. FKN was revising the manuscript critically. CL was responsible for the radio-oncological part of the study. RAA was involved in the interpretation of the results of the study and performed the statistical analysis. MAL was involved in the interpretation of the results and critically revised the manuscript. SH collected the data and helped to draft the manuscript. All authors read and approved the final manuscript.

## References

[B1] Slifer KJ (1996). A video system to help children cooperate with motion control for radiation treatment without sedation. J Pediatr Oncol Nurs.

[B2] Seiler G, De Vol E, Khafaga Y, Gregory B, Al-Shabanah M, Valmores A, Versteeg D, Ellis B, Mustafa MM, Gray A (2001). Evaluation of the safety and efficacy of repeated sedations for the radiotherapy of young children with cancer: a prospective study of 1033 consecutive sedations. Int J Radiat Oncol Biol Phys.

[B3] Lew CM, LaVally B (1995). The role of stereotactic radiation therapy in the management of children with brain tumors. J Pediatr Oncol Nurs.

[B4] Scott L, Langton F, O'Donoghue J (2002). Minimising the use of sedation/anaesthesia in young children receiving radiotherapy through an effective play preparation programme. Eur J Oncol Nurs.

[B5] Klosky JL, Tyc VL, Srivastava DK, Tong X, Kronenberg M, Booker ZJ, de Armendi AJ, Merchant TE (2004). Brief report: Evaluation of an interactive intervention designed to reduce pediatric distress during radiation therapy procedures. J Pediatr Psychol.

[B6] Zelikovsky N, Rodrigue JR, Gidycz CA, Davis MA (2000). Cognitive behavioral and behavioral interventions help young children cope during a voiding cystourethrogram. J Pediatr Psychol.

[B7] Liossi C, Hatira P (1999). Clinical hypnosis versus cognitive behavioral training for pain management with pediatric cancer patients undergoing bone marrow aspirations. Int J Clin Exp Hypn.

[B8] Loewy J, Hallan C, Friedman E, Martinez C (2005). Sleep/Sedation in children undergoing EEG testing: a comparison of chloral hydrate and music therapy. J Perianesth Nurs.

[B9] Manne SL, Bakeman R, Jacobsen PB, Gorfinkle K, Redd WH (1994). An analysis of a behavioral intervention for children undergoing venipuncture. Health Psychol.

[B10] Doverty N (1992). Therapeutic use of play in hospital. Br J Nurs.

[B11] Felt BT, Mollen E, Diaz S, Renaud E, Zeglis M, Wheatcroft G, Mendelow D (2000). Behavioral interventions reduce infant distress at immunization. Arch Pediatr Adolesc Med.

[B12] Pressdee D, May L, Eastman E, Grier D (1997). The use of play therapy in the preparation of children undergoing MR imaging. Clin Radiol.

[B13] DuHamel KN, Redd WH, Vickberg SM (1999). Behavioral interventions in the diagnosis, treatment and rehabilitation of children with cancer. Acta Oncol.

[B14] Hicks MD, Lavender R (2001). Psychosocial practice trends in pediatric oncology. J Pediatr Oncol Nurs.

[B15] Janssen F (1989). [Integration of psychosocial services into the management of the child with cancer]. Klin Padiatr.

[B16] Patenaude AF, Kupst MJ (2005). Psychosocial functioning in pediatric cancer. J Pediatr Psychol.

[B17] Schreck D, Glanzmann G, Sauter S, Niemeyer C (1994). [Support in irradiation: a responsibility of the psychosocial team]. Klin Padiatr.

[B18] Slifer KJ, Bucholtz JD, Cataldo MD (1994). Behavioral training of motion control in young children undergoing radiation treatment without sedation. J Pediatr Oncol Nurs.

[B19] Brigitte van den Heuvel CM, (DLFH) KDLFAKVD Radio-Robby und sein Kampf gegen die bösen Krebszellen.

[B20] Michael Grotzer AS, Michael Grotzer KZ (2003). Eugen und der freche Wicht.

[B21] Landolt MA, Vollrath M, Niggli FK, Gnehm HE, Sennhauser FH (2006). Health-related quality of life in children with newly diagnosed cancer: a one year follow-up study. Health Qual Life Outcomes.

